# Sociodemographic, Electrophysiological, and Biochemical Profiles in Children with Attention Deficit Hyperactivity Disorder and/or Epilepsy

**DOI:** 10.1155/2018/8932817

**Published:** 2018-11-29

**Authors:** Sameh A. Abd El Naby, Yahya M. Naguib

**Affiliations:** ^1^Paediatrics Department, Faculty of Medicine, Menoufia University, Menoufia, Egypt; ^2^Clinical Physiology Department, Faculty of Medicine, Menoufia University, Menoufia, Egypt

## Abstract

Attention deficit hyperactivity disorder (ADHD) is among the most prevalent neurobehavioral disorders affecting children worldwide. The prevalence of ADHD is higher in children with epilepsy. Despite the plethora of conducted work, the precise cause of ADHD is not identified yet. We studied here the sociodemographic, clinical, electrophysiological, and biochemical profiles of children with ADHD, epilepsy, and ADHD with epilepsy. Subjects were divided into 4 groups (25 child/group): I—control, II—ADHD, III—epilepsy, and IV—ADHD with epilepsy. Male to female ratio was significantly (*p* < 0.05) higher in the ADHD (3.1) and ADHD with epilepsy (2.1) groups when compared to the control (1.08) or epilepsy (1.08) groups. Positive family history was significantly evident in patients with epilepsy and ADHD with epilepsy, but not in the control or ADHD groups. Speech development was significantly delayed in the ADHD and ADHD with epilepsy groups. EEG abnormalities were detected in patients with ADHD (12%) and ADHD with epilepsy (68%). Focal frontal activities were significantly detectable in the ADHD (100%) and ADHD with epilepsy (77.8%) groups, whereas focal temporal activity was significantly present in the epilepsy (83.3%) group. Serum ferritin was significantly lower in the ADHD group (110.27 ± 6.64 *η*g/ml) when compared to the control (134.23 ± 14.82 *η*g/ml), epilepsy (159.66 ± 33.17 *η*g/ml), and ADHD with epilepsy (203.04 ± 50.64 *η*g/ml) groups. Serum zinc was significantly higher in the ADHD, epilepsy, and ADHD with epilepsy groups (236.63 ± 20.89, 286.74 ± 43.84, and 229.95 ± 67.34 *μ*g/dl, respectively), when compared to the control group (144.21 ± 17.40 *μ*g/dl). Serum adenosine deaminase was insignificantly different among the groups. Our results indicate that gender and family history are significant moderators in the aetiology of ADHD and epilepsy or their comorbidity. We also demonstrated that EEG could be central in the assessment of ADHD with epilepsy cases. Serum ferritin and zinc alteration may contribute significantly in ADHD and epilepsy pathophysiology.

## 1. Introduction

Attention deficit hyperactivity disorder (ADHD) is one the most prevalent childhood neuropsychiatric disorders worldwide in both developed and developing countries. ADHD affects 5–15% of school-age children and was found to persist in 30–50% of these cases through adolescence [1]. ADHD is characterized by inattention, hyperactivity, and impulsivity. These children usually experience learning difficulties and often demonstrate academic failure and underachievement [2]. ADHD can be considered as a financial load; the high levels of unemployment and turnover of jobs associated with ADHD in adults can lead to lower household income levels. Additionally, parents with ADHD children may suffer financially as a result of reduced work efficiency caused by managing their child's condition. ADHD also represents a national burden as a consequence of the increased use of ADHD-associated healthcare resources [3].

Epilepsy is a common chronic neurological disorder characterized by seizures. The annual incidence of epilepsy is estimated to be around 40–70/100,000 births/year in developed countries and 100–190/100,000 births/year in developing countries [4]. Although in many epileptic cases a precise cause cannot be identified, several factors such as brain trauma, strokes, brain cancer, and drug and alcohol misuse have been suggested. It has been reported previously that the prevalence of ADHD is higher in children with epilepsy than in the general paediatric population ranging widely between 8 and 77% [5]. Seizure is a cofactor among children with epilepsy that can induce hyperactivity, inattention, and impulsivity. Epileptic seizures may result from abnormal, excessive, or hypersynchronous neuronal activity in the brain. Substantial data suggest that seizure control could improve the symptoms of ADHD [6].

Although several studies have addressed the neurobiology of ADHD from imaging and genetics points of view, relatively inadequate attention has been given to study the factors or mechanisms that may be involved. In the present investigation, we studied the sociodemographic and electrophysiological data, as well as the serum levels of some biomarkers in children with ADHD and ADHD with epilepsy in comparison to those in control healthy and epileptic volunteers.

## 2. Subjects and Methods

100 children of comparable age were recruited for the present cross-sectional study. Patients were selected from those attending the Paediatrics Neurology Clinic in Menoufia University Hospital and those admitted to the Paediatrics Department in Suzanne Mubarak Hospital, as well as 25 healthy volunteers who served as controls. 75 selected patients were diagnosed with ADHD, epilepsy, or comorbid ADHD and epilepsy. All the controls and patients provided written informed consents following a comprehensive description of the study. The Ethics Committee of the Menoufia University Faculty of Medicine approved the study. A semistructured form was used to detect several sociodemographic and clinical variables of the patients, and the medical records of the patients were reviewed.

Subjects were divided into 4 groups (25 child/group): I—control group, II—ADHD group (diagnosed according to the ADD/ADHD DSM IV-Based Diagnostic Screening and Rating Scale), III—epilepsy group (diagnosed according to the International League Against Epilepsy (ILAE) classification), and IV—ADHD with epilepsy group (diagnosed according to the ADD/ADHD DSM IV-Based Diagnostic Screening and Rating Scale, ILAE, and EEG).

Patients with a history of chronic systemic diseases such as diabetes mellitus and hypertension, other psychological or neurological disorders, ADHD criteria for less than 6 months, uncontrolled epilepsy, or with IQ < 70 were excluded. None of the children were taking nutritional supplements such as antioxidants. All subjects underwent brain magnetic resonance imaging (MRI) evaluation, and all were free of any abnormalities. Delayed speech was assessed by both history taking from the parents and preschool language scale 5 (PSL-5).

### 2.1. Blood Sample Collection

Whole venous blood was collected via EDTA-lined Vacutainer tubes. The blood was allowed to coagulate for 30 minutes at room temperature. Blood samples were then centrifuged at 2000 rpm for 15 minutes to separate serum samples. Serum was then separated and stored at −80°C. The serum ferritin (*η*g/ml) and adenosine deaminase (ADA) (U/l) levels were determined by in vitro enzyme-linked immunosorbent assay kits (Aviva Systems Biology Corporation, USA), using an automatic optical reader (SUNRISE Touchscreen, TECHAN, Salzburg, Austria) [8, 9]. The serum zinc level was determined using commercially available kits (QuantiChrom™, BioAssay Systems, USA) as described previously [10]. Results were expressed as *μ*g/dl. Assay was determined by routine kinetic and fixed-rate colorimetric methods on a Jenway Genova autoanalyser (UK).

### 2.2. Statistical Analysis

Results are expressed as mean ± standard error (SE). Student's *t*-test was used for the comparison between the means of any 2 groups of patients, while Pearson's chi-square (*χ*^2^), Kruskal-Wallis (K), and Fisher's exact tests were used for the comparison between qualitative variables using Statistical Package for Social Sciences (SPSS) software and the probability of chance (*p* values). *p* values < 0.05 were considered significant.

## 3. Results

The sociodemographic profiles of the studied groups are illustrated in Table 1. The study included 25 control subjects, 25 patients with ADHD, 25 patients with epilepsy, and 25 patients with ADHD and epilepsy. The mean ages of the control, ADHD, epilepsy, and AHHD with epilepsy groups were 5.66 ± 3.99, 4.0 ± 2.47, 4.25 ± 1.79, and 4.11 ± 1.72 years, respectively, with no significant differences (*p* > 0.05) between the studied groups. Gender distribution in the ADHD and ADHD with epilepsy groups showed significant increased male numbers (19 males and 6 females and 17 males and 8 females, respectively, *p* < 0.05), when compared to the corresponding values in the control (13 males and 12 females) or epilepsy (13 males and 12 females) groups. There was insignificant difference (*p* > 0.05) in gender when comparing the corresponding values in the control and epilepsy groups or when comparing the values in the ADHD and ADHD with epilepsy groups. Positive family history of seizures or neurological diseases was significantly different (*p* < 0.05) in the epilepsy and ADHD with epilepsy groups when compared to the corresponding values in the control and ADHD groups (16 and 18 vs 2 and 3, respectively). However, it was insignificantly different in the ADHD group when compared to the corresponding value in the control group (*p* > 0.05). There was insignificant difference in consanguinity or developmental history among the studied groups (*p* > 0.05).

Speech development was significantly delayed in the ADHD (15 patients, 60%) and ADHD with epilepsy (15 patients, 60%) groups, when compared to the corresponding values in the control (2 subjects, 8%) or epilepsy (3 patients, 12%) groups. There was insignificant difference between epileptics and control subjects and also between the ADHD and ADHD with epilepsy patients (Table 2).

The distribution of the different subtypes of ADHD in the ADHD and ADHD with epilepsy groups is shown in Table 3. The number of children with inattention subtype (ADHD-I) was significantly higher in the ADHD with epilepsy group (15 patients, 60%), when compared to the ADHD group (3 patients, 12%). On the contrary, the number of patients with the combined inattention and hyperactive (ADHD I/H) subtype was significantly higher in the ADHD group (17 patients, 68%), when compared to the corresponding value in the ADHD with epilepsy group (5 patients, 20%). There was insignificant difference in the number of patients with the hyperactive subtype (ADHD-H) between both groups.

Table 4 demonstrates different seizure types and subtypes in the epilepsy and ADHD with epilepsy groups. There was insignificant difference (*p* > 0.05) between the epilepsy and ADHD with epilepsy groups regarding the types of seizures (22 generalized and 3 partial vs 21 generalized and 4 partial). However, there was an important difference in the number of patients with generalized myoclonic convulsion which was significantly higher in the ADHD with epilepsy group (5 patients, 23.8%) when compared to the corresponding value in the epilepsy group (0 patients). The number of patients with complex partial convulsions in the ADHD with epilepsy group was 4 (100%), which was significantly higher when compared to the corresponding value in the epilepsy group (0 patients), while the number of patients with focal secondary generalization convulsions in the ADHD with epilepsy group (0 patients) was significantly lower when compared to that in the epilepsy group (3 patients, 100%).

**Figure 1:**
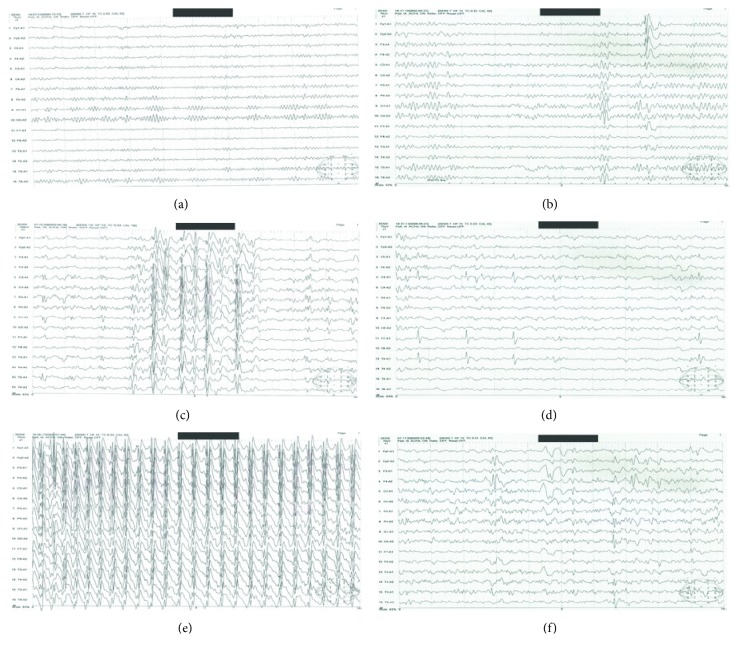
Representative traces of EEG in the studied groups: (a) control group; (b) focal frontal activity in the ADHD group (3/3); (c) generalized activity in the epilepsy group (7/13); (d) focal temporal activity in the epilepsy group (5/13); (e) generalized activity in the ADHD + epilepsy group (8/17); (f) focal frontal activity in the ADHD + epilepsy group (7/17). *n*/*N* = number of cases/total number of patients with abnormal EEG; total number of subjects—25/group.

As shown in Table 5 and Figure 1, the number of patients with abnormal EEG in the ADHD group (3 patients, 12%) was insignificantly different when compared to the corresponding value in the control group (0 subjects). On the other hand, the number of patients with abnormal EEG in the epilepsy group (13 patients, 52%) and ADHD with epilepsy group (17 children, 68%) was significantly higher when compared to the corresponding values in the control or ADHD groups. There was insignificant difference between the epilepsy and ADHD with epilepsy groups. There was insignificant difference in the types of EEG abnormality between the studied groups. However, the site of focal activities showed significant differences. The number of patients with EEG showing frontal lobe epileptogenic activity was significantly higher in the ADHD group (3 patients, 100%) and ADHD with epilepsy group (7 patients, 77.8%), when compared to the epilepsy group (0 patients). Temporal lobe epileptogenic activity in the epilepsy group (5 patients, 83.3%) was significantly higher when compared to the corresponding values in the ADHD (0 patients) or ADHD with epilepsy (1 patient, 11.1%) groups. There was insignificant difference between the ADHD and ADHD with epilepsy groups in the site of epileptogenic activities, and there was also insignificant difference in the number of patients with EEG showing occipital or parietal lobe epileptogenic activity among the studied groups.

**Figure 2:**
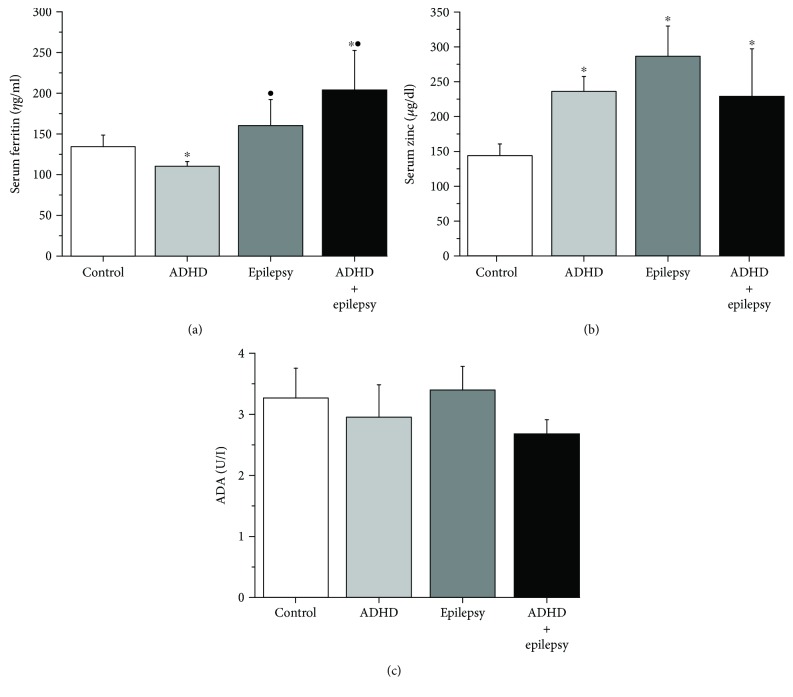
Serum biochemical profiles in ADHD and/or epilepsy: (a) ferritin (*η*g/ml) in the control (white column), ADHD (light-grey column), epilepsy (dark-grey column), and ADHD + epilepsy (grey column) groups; (b) zinc (*μ*g/dl) in the control (white column), ADHD (light-grey column), epilepsy (dark-grey column), and ADHD + epilepsy (grey column) groups; (c) ADA (U/l) in the control (white column), ADHD (light-grey column), epilepsy (dark-grey column), and ADHD + epilepsy (grey column) groups. ^∗^Significant when compared to the control group. ^●^Significant when compared to the ADHD group. Significant = *p* < 0.05; number of subjects—25/group.

The serum ferritin level in patients with ADHD (110.27 ± 6.46 *η*g/ml) was significantly lower when compared to those in the control (134.23 ± 14.82 *η*g/ml), epilepsy (159.66 ± 33.17 *η*g/ml), or ADHD with epilepsy (203.04 ± 50.64 *η*g/ml) groups. Serum ferritin level was significantly higher in the ADHD with epilepsy group when compared to the control subjects (Figure 2(a)). The mean value of serum Zn was significantly higher in the ADHD (236.63 ± 20.89 *μ*g/dl), epilepsy (286.74 ± 43.84 *μ*g/dl), and ADHD with epilepsy (229.95 ± 67.34 *μ*g/dl) groups, when compared to the corresponding value in the control group (144.21 ± 17.40 *μ*g/dl). There was insignificant difference in serum Zn between the ADHD, epilepsy, and ADHD with epilepsy groups (Figure 2(b)). The serum level of ADA was insignificantly different between the control (3.26+ 0.50 U/l), ADHD (2.95 ± 0.55 U/l), epilepsy (3.39 ± 0.40 U/l), and ADHD with epilepsy (2.68 ± 0.23 U/l) groups (Figure 2(c)). There was a significant positive correlation (*p* < 0.05) between serum ADA and family history of neurological diseases in the control group. There was also a significant positive correlation between serum zinc and family history of neurological diseases and between serum ferritin and the age in the epilepsy group (Table 6). There were insignificant relations between serum markers and the sociodemographic data in the ADHD and ADHD with epilepsy groups (data not shown).

## 4. Discussion

ADHD is considered one of the most widespread disorders in school-age children. Generally, ADHD is characterized by persistent patterns of inattention, hyperactivity, and impulsivity. Childhood-onset ADHD was long thought to be resolved upon maturation; however, follow-up studies documented the persistence of ADHD into adulthood. ADHD affects the development of children which may lead to academic difficulties and social problems, and it has significant impact on family members, peers, and teachers. Children with epilepsy are frequently presented with comorbid ADHD. ADHD has not been attributed to a specific aetiology, and several findings support a multifactorial hypothesis. Studies which addressed the neurobiology of ADHD relied mainly on imaging and genetics; however, little attention has been given to other possible mechanisms such as organic brain diseases, metabolic dysfunction, and the immune system response. The present investigation was conducted to study the electrophysiological and biochemical changes in children with ADHD in relation to epilepsy.

In the present study, the male-to-female ratio was significantly higher in the ADHD and ADHD with epilepsy groups when compared to the control or the epilepsy groups. Similar findings were reported previously [11]. However, it was argued that the male-to-female ratio in ADHD is higher in clinic samples (up to 10 : 1) but not that in community samples (2 : 1 to 4 : 1), which was explained by referral bias rather than a biological mechanism [12, 13]. Positive family history of seizures and neurological diseases was significantly higher in the epilepsy and ADHD with epilepsy groups when compared to the control or ADHD groups. Our results were in consistency with previously published reports [14]. Epileptogenesis has been linked to a number of genetic disorders. Many epilepsy-related genes have been identified; each one may determine different aspects of the phenotype, including age of onset, seizure type, and seizure focus [15]. We could assume then that gender may be a central moderator if suspecting ADHD or ADHD with epilepsy, whereas family history may underlie epilepsy or epilepsy with ADHD.

In the present work, speech development was significantly delayed in the ADHD group when compared to the control group. Similar results were reported previously [16]. This could be explained by the impairment of the working memory abilities; abnormalities in cortical, basal ganglia, and cerebellar brain regions; and auditory brain stem dysfunction. At the brain stem level, children with ADHD have a common processing dysfunction of nonspeech and speech stimuli [17]. Interestingly, we found that speech development was only delayed when epilepsy and ADHD coexisted, and not in the stand-alone cases of epilepsy. Epilepsy-ADHD comorbidity may depend on the duration of epilepsy and seizure frequency [5]. Learning, behavioral, speech, and language difficulties were linked to certain types of epilepsy such as Landau Kleffner syndrome, Tassinari syndrome, and Lennox-Gastaut syndrome, especially if they occurred in the first 24 months after birth [18].

In the present study, the most prevalent ADHD subtype in the ADHD group was the combined form (I/H), while the inattention subtype was the most prevalent in the ADHD with epilepsy group. Our results were in agreement with several published reports [5]. ADHD is a neurobehavioral disorder that results from interruption of frontal-striatal brain networks. Disruption of these brain networks, by seizures or structural brain lesions, can result in ADHD-like symptoms or behavior problems [19]. Patients with early-onset, more frequent, and/or more severe seizures are at greater risk of developing ADHD. This can be explained by possible alteration of the development of the frontal brain networks during childhood. Some antiepileptic drugs (AEDs) have undesirable side effects in the form of inattention or ADHD-like symptoms [20]. It has been reported that children with complex partial seizures or benign epilepsy with centrotemporal spikes (BECTS) may struggle significantly with sustained attention. Also, right-sided interictal epileptiform activity in these children interfered with right hemispheric activities including attention [5]. We showed here that the generalized tonic-clonic seizures were the most prevalent type in both the epilepsy and ADHD with epilepsy groups. There was significant increase in the number of cases with generalized myoclonic seizures in the ADHD with epilepsy patients. Epileptics with generalized myoclonic seizures such as juvenile myoclonic epilepsy have demonstrated abnormal frontal lobe gray matter volumes and cognitive impairment [21, 22].

EEG is usually normal in most cases with ADHD; therefore, it was expected to find insignificant EEG changes in the ADHD group when compared to controls. But interestingly, three cases in the ADHD group showed abnormal focal epileptogenic activity in the frontal lobe in their EEGs despite negative previous history of seizures. This was consistent with previous reports [23]. EEG epileptiform changes have been found in 6.1% of 347 subjects with ADHD without epilepsy [24]. A principal relationship between subclinical epileptic waves and occurrence of ADHD symptoms is raised. In previous reports, treatment of such activities with AEDs led to recovery from ADHD symptoms [25]. Accordingly, it is recommended to perform EEG when patients with ADHD present with history of previous seizures, prenatal events, history of head trauma, variable behavioral manifestations, other existing cognitive disorders such as mental retardation, or positive family history of seizures. 52% of epileptics in the current study had EEG abnormality. Nevertheless, normal EEG results were found in half of the children who have epileptic seizures. Interestingly, children who did not have epileptic seizures could be presented with genetic epileptic-form abnormalities in their EEG [26]. Taken together, normal EEG results do not exclude the diagnosis of epilepsy and epileptogenic discharges in EEG are not a precise diagnosis of the disease. It was found not only that the number of children with abnormal EEG in the ADHD with epilepsy group was significantly higher when compared to the corresponding value in the control group but also that the percentage of frontal lobe epileptogenic activity was significantly higher in ADHD with epilepsy patients when compared to those with epilepsy only. The risk of ADHD increases in children whose seizures are more resistant and whose EEGs show evident epileptiform discharge [27]. In some epilepsy syndromes, ADHD is considered a high risk factor; 67% of the patients with frontal lobe epilepsy and 61% with childhood absence epilepsy (CAE) had comorbid ADHD [25]. Children with frontal lobe epilepsy had lower scores in working memory and processing when compared to children with temporal lobe seizures. In the same study, there was more impairment in executive functioning in children with idiopathic generalized epilepsy (IGE) than in children with idiopathic localization-related epilepsy [28]. Frontal-striatal network dysfunction is related to ADHD without epilepsy whereas evidence of frontal lobe dysfunction appears in both focal-onset and the generalized-onset types of epilepsy [29].

We demonstrated here that the mean value of the serum ferritin level was significantly lower in the ADHD group, while on the contrary, the serum ferritin level in the ADHD with epilepsy group was significantly higher when compared to the corresponding values in the control or epilepsy groups. Similar results were reported previously, where the low serum ferritin level was associated with hyperactivity symptoms in children with ADHD [30]. Several studies highlighted the role of the low serum ferritin level in the pathogenesis of ADHD. Most revealed that dopamine was a key element in ADHD pathophysiology. The association between ADHD and the genes regulating dopamine, norepinephrine, serotonin, and gamma-aminobutyric acid (GABA) has been reported. Dopamine may play a central role because of its association with the modulation of psychomotor activity and executive functions, which are the main clinical features of individuals with ADHD [31]. Iron is a cofactor of tyrosine hydroxylase, the rate-limiting enzyme for dopamine synthesis. Therefore, brain iron stores may influence dopamine synthesis and subsequently affect various behavioral features, particularly those described in people with ADHD. Iron serves as a cofactor in the synthesis of important neurotransmitters such as dopamine, norepinephrine, and serotonin, and deficiency in early years of life can negatively affect neural and behavioral development [32]. Iron supplementation (ferrous sulphate, 80 mg/day for 12 weeks) for ADHD children was associated with significant progressive decrease in the ADHD rating scale [30]. Supporting our results, previously published data reported insignificant difference in serum ferritin levels between epileptic patients and controls [33]. Nevertheless, prolonged seizures can disrupt the blood brain barrier and result in extravasation of blood and break down of red blood cells and haemoglobin. Iron liberated from haemoglobin and haemoglobin itself are associated with the generation of ROS and subsequent cell death, so increased serum ferritin may accompany oxidative stress [34]. This may explain how elevated serum ferritin level in patients with ADHD and epilepsy may contribute in the pathogenesis of the disease.

Serum zinc (Zn) levels were significantly higher in the ADHD, epilepsy, and ADHD with epilepsy groups, when compared to the corresponding value in control group. Zn homeostasis is necessary for the proper development of the brain especially for the cerebellum, stellate, and basket and also for Purkinje and granule cells [35]. Zn may have a second messenger activity in the brain; in mammals, Zn-containing vesicles were found mostly in the glutamatergic neurons in the brain cortex, hippocampus, amygdala, and brain compartments responsible for learning, memory, cognition, and mood regulation [36]. Zn and glutamate are released from the synaptic vesicles into the synaptic cleft during neuronal activity. Zn interacts with the postsynaptic receptors leading to inhibition of the GABA receptors and resulting in increased neuronal excitability [37]. Persistent exposure to Zn may explain the elevated serum levels of Zn. At the present, people are being exposed to a range of Zn-containing organic and inorganic materials such as food supplements and additives, medicines, disinfectants, antiseptic, deodorant preparations, and dental cement [38]. Zn intake in the range of approximately 100–300 mg/daily (doses likely to induce chronic toxicity) is common among people using Zn-containing supplements and oral Zn medicines. Prolonged Zn exposure via these routes has been associated with copper deficiency. The antioxidant enzyme copper-zinc superoxide dismutase (Cu-Zn-SOD) is thought to be very sensitive to changes in the plasma Zn/Cu ratio, and therefore, alterations in the SOD activity with Zn supplementation may result in excess free radicals that could damage the cell membrane [39]. Studies also reported competitive interactions between Zn and iron which may result in decreased serum ferritin [38]. We could then suggest that the high serum Zn level may had led to hypoferritinemia, copper deficiency, or oxidative stress; all or any of them may had contributed to the pathogenesis of ADHD. Alteration in Zn homeostasis in the brain has been associated with epilepsy. However, it was not determined if Zn was a cause or a consequence of seizures [40]. Several minerals are vital for the normal function of the central nervous system. Changes in body electrolytes such as sodium, potassium, magnesium, and trace elements such as copper and Zn may trigger convulsions and subsequently epilepsy. Zn potentiates glycine-mediated currents and regulates voltage-gated calcium, sodium, potassium, and chloride channels. Hence, Zn can modulate neuronal excitability under normal conditions. Nevertheless, elevated Zn level can be neurotoxic and may contribute to the neuropathology associated with a variety of conditions, such as seizures, Alzheimer's disease, and stroke [41]. Antiepileptic drugs may alter the serum levels of Zn. Valproic acid-treated patients had significantly higher levels of serum Zn [42, 43].

In the present investigation, serum levels of ADA were insignificantly different among the studied groups. ADA is an essential enzyme for the T lymphocyte maturation and function. As the plasma activity of ADA has been suggested to be increased in inflammatory diseases, ADA has been considered as an indicator of cell-mediated immunity [44]. Streptococcal infections are very frequent during childhood, and they may occasionally result in neurologic and/or psychiatric symptoms [45]. Our results were in disagreement with a previously published paper reporting an increase in serum ADA in children with ADHD which was suggested to play a role in the pathogenesis of the disease [46]. Our results may have differed due to the dissimilarity in the age of the samples (4 ± 1.79 years in our study vs 10 ± 2.4 years in the before-mentioned study). Age could be an important factor in immune system sensitization; hence, the older the child is, the more is the possibility that he/she had encountered infections. Ferritin itself could be considered as an inflammatory marker and a leakage product from cell damage [47]. The decreased ferritin level in the ADHD group in the present study supports the assumption that the contribution of the immune system in the pathogenesis of ADHD may not be evident at early stages of the disease. Our results were also in disagreement with the accumulating evidence suggesting that inflammatory and immune reactions may contribute to the increased neuronal excitability and decreased seizure threshold and therefore are likely to be involved in epileptogenesis [48]. A decrease in adenosine deamination was observed after 20 min of successive pentylenetetrazol-induced seizures in zebra fish, suggesting a modulation of extracellular adenosine levels in the occurrence of repetitive seizures [49]. Albeit, this could also be explained by the difference in species or whether ADA was assessed in the serum or tissue samples.

## 5. Conclusion

To our knowledge, this is the first paper to study a full spectrum of neurobehavioral disorders which can exist together. Our attempt to characterize ADHD, epilepsy, and their comorbidity is aimed at elucidating common or diverse underlying mechanisms. Gender and family history were shown to be key moderator factors. EEG, although not a precise diagnostic tool, should be considered if ADHD is suspected especially if the patients are positive for family history of seizures, prenatal events, history of trauma, or coexistence of other behavioral manifestations. Iron and zinc metabolism may contribute to the pathophysiology of ADHD, epilepsy, or their comorbidity. Accordingly, serum biomarkers may play a promising role in early identification of the diseases.

## 6. Study Limitations

The sample size of the study was relatively small, and more work including larger numbers of the study children should continue. More work on the correlation between risk factors is essential.

## Figures and Tables

**Table 1:** Sociodemographic characteristics.

Sociodemographic data	Control (*N* = 25)		ADHD (*N* = 25)		Epilepsy (*N* = 25)		ADHD with epilepsy (*N* = 25)		Total (*N* = 100)		Test	*p* value
	No.	%	No.	%	No.	%	No.	%	No.	%	K	
*Age (years)*												
Mean ± SD	5.66 ± 3.9	9	4.00 ± 2.47		4.25 ± 1.79		4.11 ± 1.72				2.14	0.10

											**χ** ^2^	
*Gender*											3.13^1^	**0.02** ^∗^
Male	13	52.0	19	76.0	13	52.0	17	68.0	62	62.0	0.00^2^	1.00
Female	12	48.0	6	24.0	12	48.0	8	32.0	38	38.0	1.33^3^	**0.04** ^∗^
Male : female ratio		1.08		3.1		1.08		2.1			3.13^4^	**0.02** ^∗^
											0.40^5^	0.53
											1.33^6^	**0.04** ^∗^

*Family history of neurological disorders*											0.22 ^1^	0.63
											14.67^2^	**0.001** ^∗^
Positive	2	8.0	3	12.0	16	64.0	18	72.0	39	39.0	21.33^3^	
Negative	23	92.0	22	88.0	9	36.0	7	28.0	61	61.0	14.35^4^	**0.001** ^∗^ **0.001** ^∗^
											18.47^5^	**0.001** ^∗^
											0.37 ^6^	0.54

*Consanguinity*											0.00 ^1^	1.00
Positive	3	12.0	3	12.0	7	28.0	5	20.0	18	18.0	1.13^2^	0.29
Negative	22	88.0	22	88.0	18	72.0	20	80.0	82	82.0	0.15 ^3^	0.69
											2.00 ^4^	0.16
											0.60 ^5^	0.44
											0.44 ^6^	0.51

*Developmental history*											0.17 ^1^	0.68
											0.14 ^2^	0.7^1^
Normal	21	84.0	22	88.0	20	80.0	18	72.0	81	81.0	1.05 ^3^	0.30
Delayed	4	16.0	3	12.0	5	20.0	7	28.0	19	19.0	0.60 ^4^	0.44
Lost after being acquired	0	0.0	0	0.0	0	0.0	0	0.0	0	0.0	2.00 ^5^	0.16
											0.44 ^6^	0.51

^1^ADHD vs control. ^2^Epilepsy vs control. ^3^ADHD + epilepsy vs control. ^4^Epilepsy vs ADHD. ^5^ADHD + epilepsy vs ADHD. ^6^ADHD + epilepsy vs epilepsy. K: Kruskal-Wallis test. *χ*^2^: Pearson's chi-square test. ^∗^Significant (*p* < 0.05).

**Table 2:** Speech development among the studied groups.

Speech development	Control (*N* = 25)		ADHD (*N* = 25)		Epilepsy (*N* = 25)		ADHD with epilepsy (*N* = 25)		Total (*N* = 100)		*χ* ^2^	*p* value
	No.	%	No.	%	No.	%	No.	%	No.	%		
Normal	23	92.0	10	40.0	22	88.0	10	40.0	65	65.0	15.06^1^	**0.001** ^∗^
											0.22^2^	**0.63**
											15.06^3^	0.63
											12.50^4^	**0.001** ^∗^
											15.06^1^	**0.001** ^∗^
											12.50^4^	
Delayed	2	8.0	15	60.0	3	12.0	15	60.0	35	35.0		
											0.00^5^	1.00
											12.50^6^	**0.001** ^∗^

^1^ADHD vs control. ^2^Epilepsy vs control. ^3^ADHD + epilepsy vs control. ^4^Epilepsy vs ADHD. ^5^ADHD + epilepsy vs ADHD. ^6^ADHD + epilepsy vs epilepsy. *χ*^2^: Pearson's chi-square test. ^∗^Significant (*p* < 0.05).

**Table 3:** Distribution of ADHD subtypes in the ADHD and ADHD with epilepsy groups.

Types of ADHD	ADHD (*N* = 25)		ADHD with epilepsy (*N* = 25)		Total (*N* = 50)		*χ* ^2^	*p* value
	No.	%	No.	%	No.	%		
ADHD/I (inattention)	3	12	15	60	18	36.0	12.50	**0.001** ^∗^
ADHD/H (hyperactive)	5	20	5	20	10	20.0	0.00	1.00
Combined ADHD (I/H)	17	68	5	20	22	44.0	11.89	**0.001** ^∗^

*χ* ^2^: Pearson's chi-square test. ^∗^Significant (*p* < 0.05).

**Table 4:** Different seizure types in the epilepsy and ADHD with epilepsy groups.

Type of seizure	Epilepsy (*N* = 25)		ADHD + epilepsy (*N* = 25)		Total (*N* = 50)		Test	*p* value
	No.	%	No.	%	No.	%	Fisher's exact	
Generalized	22	88.0	21	84.0	43	86.0	0.17	0.68
Partial	3	12.0	4	16.0	7	14.0		

*Types of generalized seizures*							*χ* ^2^	
Tonic clonic	12	54.5	8	38.1	20	46.5	1.17	0.28
Clonic	5	22.7	3	14.3	8	18.6	0.51	0.47
Tonic	2	9.1	2	9.5	4	9.3	0.23	0.63
Myoclonic	0	0	5	23.8	5	11.6	5.93	**0.01** ^∗^
Absence	0	0	3	14.3	3	7.0	3.38	0.06
Infantile spasm	0	0	0	0	0	0	—	—
Mixed	0	0	0	0	0	0	—	—
Atonic	3	13.7	0	0	3	7.0	3.08	0.07
Total	22	100	21	100	43	100	—	—

*Types of partial seizures*							*Fisher's exact*	
Complex	0	0	4	100	4	57.1	7.00	**0.008** ^∗^
Focal with secondary generalization	3	100	0	0	3	42.9		
Total	3	100	4	100	7	100		

*χ* ^2^: Pearson's chi-square test. ^∗^Significant (*p* < 0.05).

**Table 5:** EEG findings in the studied groups.

	Control (*N* = 25)		ADHD (*N* = 25)		Epilepsy (*N* = 25)		ADHD with epilepsy (*N* = 25)		Total (*N* = 100)		Test	*p* value
	No.	%	No.	%	No.	%	No.	%	No.	%		
*EEG findings*											**χ** ^2^	
Normal	25	100	22	88.0	12	48.0	8	32.0	67	67.0	3.19^1^	0.07
											17.57^2^	**0.001** ^∗^
											25.76^3^	**0.001** ^∗^
											9.19^4^	**0.002** ^∗^
Abnormal	0	0.0	3	12.0	13	52.0	17	68.0	33	33.0		
											16.33^5^	**0.001** ^∗^
											1.33^6^	0.25

*Type of abnormality*											*Fisher's exact*	
Focal	—	—	3	100	6	46.2	9	52.9	18	54.5	2.87^4^	0.09
											0.80^5^	0.37
											0.14 6	0.71
Generalized	—	—	0	0.0	7	53.8	8	47.1	15	45.4		
Total	—	—	3	100	13	100	17	100	33	100		

*Site of focal abnormality*											**χ** ^2^	
Frontal	—	—	3	100	0	0.0	7	77.8	10	55.5	9.00^4^	**0.01** ^∗^
											0.80^5^	0.67
											11.53^6^	**0.009** ^∗^
Occipital	—	—	0	0.0	1	16.7	0	0.0	1	5.6		
Parietal	—	—	0	0.0	0	0.0	1	11.1	1	5.6		
Temporal	—	—	0	0.0	5	83.3	1	11.1	6	33.3		
Total	—	—	3	100	6	100	9	100	18	100		

^1^ADHD vs control. ^2^Epilepsy vs control. ^3^ADHD + epilepsy vs control. ^4^Epilepsy vs ADHD. ^5^ADHD + epilepsy vs ADHD. ^6^ADHD + epilepsy vs epilepsy. *χ*^2^: Pearson's chi-square test. ^∗^Significant (*p* < 0.05).

**Table 6:** Relation between serum markers and sociodemographic data.

Group	Serum marker	Sociodemographic data		Test	*p* value
		Family history		Mann-Whitney	
*Control*	*ADA*	Positive (*N* = 2)	Negative (*N* = 23)	20	**0.010** ^∗^
		6.79 ± 6.74	2.90 ± 0.001		
*Epilepsy*	*Zinc*	Positive (*N* = 16)	Negative (*N* = 9)	8	**0.025** ^∗^
		351.2 ± 191.8	190.0 ± 56.0		
		*Age*			
*Epilepsy*	*Ferritin*	4.25 ± 1.97		0.520	**0.047** ^∗^

^∗^Significant (*p* < 0.05).

## Data Availability

The data used to support the findings of this study are included within the article.
